# Supplementary figures and images for: Rifampicin and isoniazid drug resistance among patients diagnosed with pulmonary tuberculosis in southwestern Uganda
Source: PLoS One. 2021 Oct 29;16(10):e0259221. doi: 10.1371/journal.pone.0259221 (PMC8555815; doi:10.1371/journal.pone.0259221)

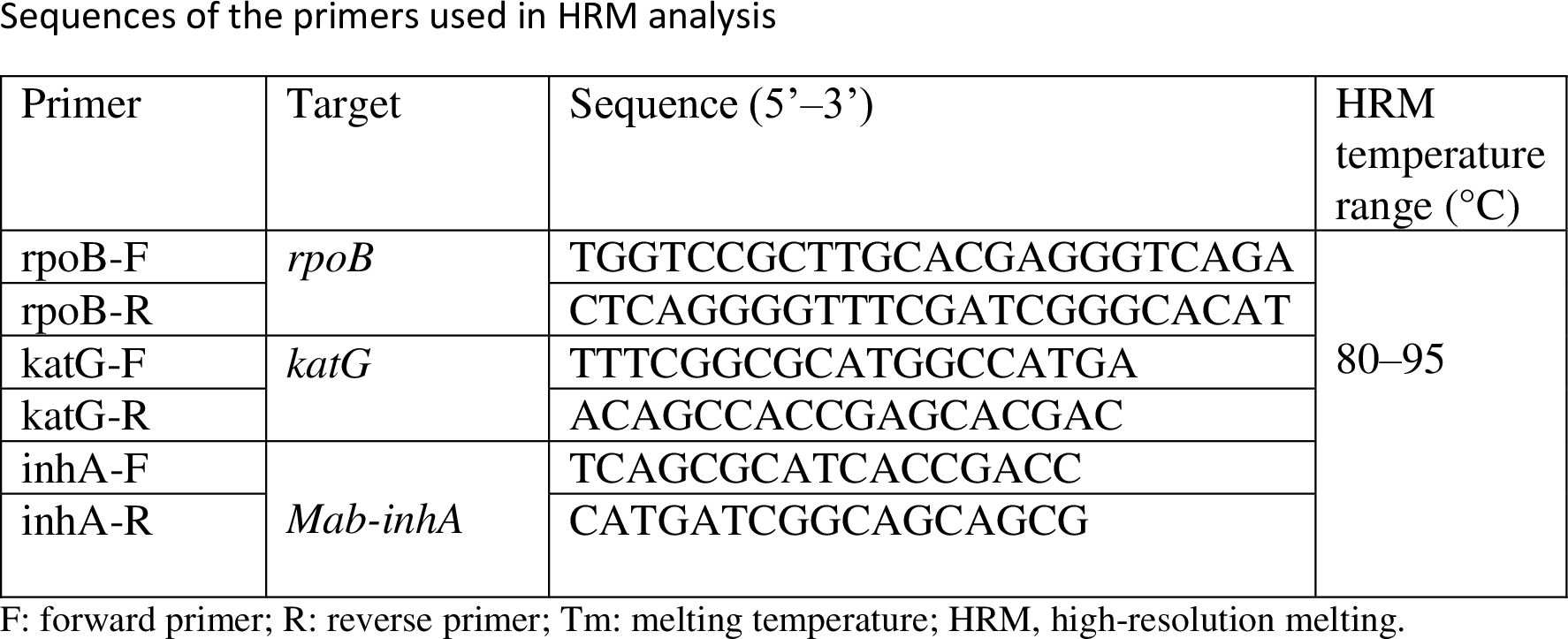

Supplement: S1 Table — (TIF) [file pone.0259221.s001.tif]

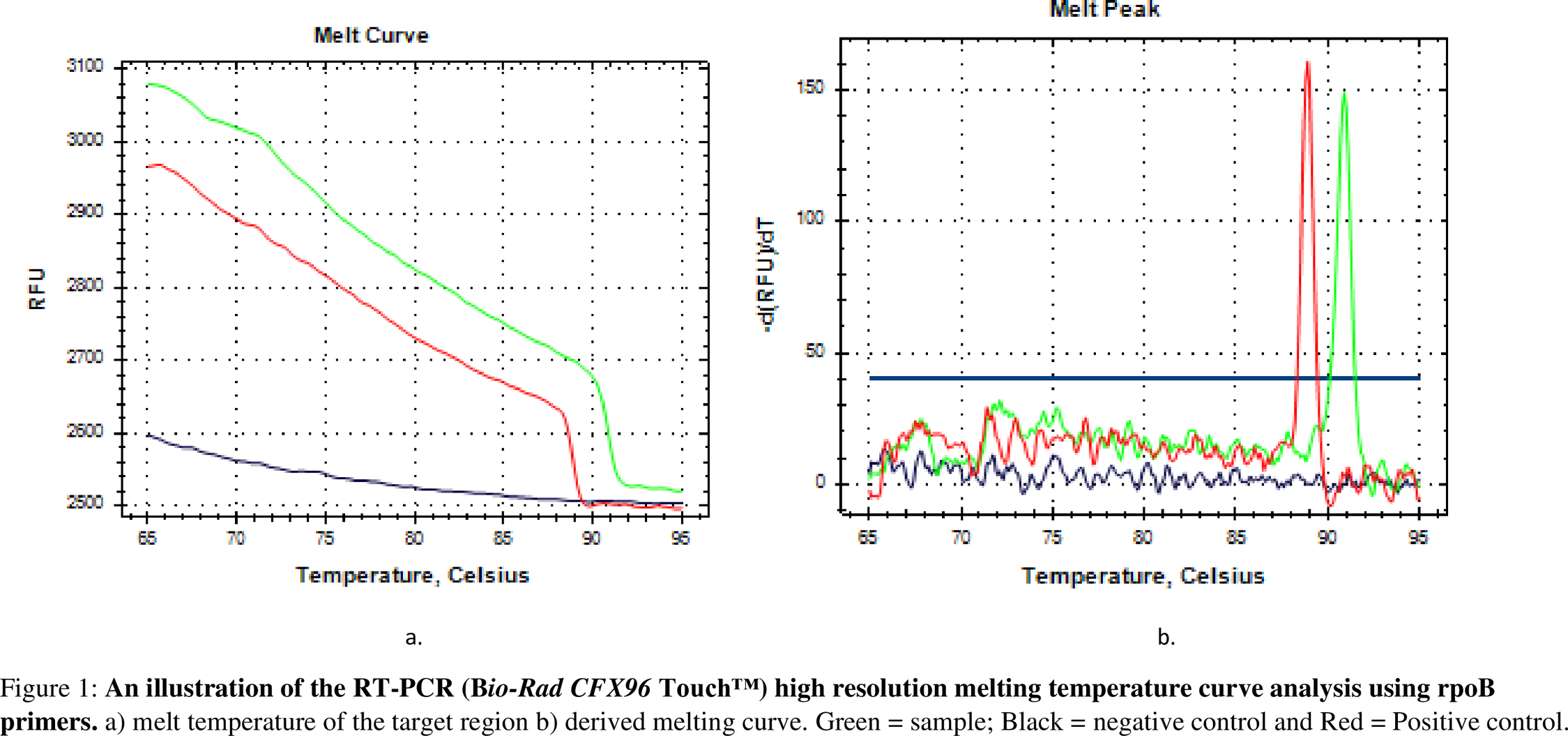

Supplement: S1 Fig — a) melt temperature of the target region b) derived melting curve. Green = sample; Black = negative control and Red = Positive control. (TIF) [file pone.0259221.s002.tif]
