# Supplementary material for: Rifampicin and isoniazid drug resistance among patients diagnosed with pulmonary tuberculosis in southwestern Uganda
Source: PLoS One. 2021 Oct 29;16(10):e0259221. doi: 10.1371/journal.pone.0259221 (PMC8555815; doi:10.1371/journal.pone.0259221)
Supplement: Attachment — Submitted filename: Response to reviewers-4.docx [file pone.0259221.s003.docx]

**REBUTTAL LETTER**

**Ref:** PONE-D-21-11660

**Title:** Rifampicin and Isoniazid drug resistance among patients diagnosed with pulmonary tuberculosis in southwestern Uganda

Dear, Dr. Christophe Sola,

Academic Editor, PLOS ONE

We appreciate the editor's and reviewers' time in assessing our manuscript. We definitely benefited from the feedback we received that helped to improve this manuscript. We evaluated each comment and made changes on the revised manuscript in order to address the concerns. All the authors were involved in the revision process, and they read and approved the final version that has been submitted. The following is a detailed response (in red) to the editor and the reviewer's comments;

**Academic Editor:**

1. Please ensure that your manuscript meets PLOS ONE's style requirements, including those for file naming. The PLOS ONE style templates can be found at

<https://journals.plos.org/plosone/s/file?id=wjVg/PLOSOne_formatting_sample_main_body.pdf> and https://journals.plos.org/plosone/s/file?id=ba62/PLOSOne_formatting_sample_title_authors_affiliations.pdf

We have checked the template and made necessary adjustments to meet the journal requirements.

 2. In your Methods section, please provide additional information about the participant recruitment method and the demographic details of your participants. Please ensure you have provided sufficient details to replicate the analyses such as:
 - a description of any inclusion/exclusion criteria that were applied to participant recruitment
- a statement as to whether your sample can be considered representative of a larger population
- a description of how participants were recruited.

Lines 90-93 provides information on the inclusion/exclusion criteria while the sample size was calculated using the Cochran sampling approach as indicted in lines 87-88. Lines 94-99, provides a description of how the patients in this study were recruited.

3. Please include additional information regarding the survey or questionnaire used in the study and ensure that you have provided sufficient details that others could replicate the analyses. For instance, if you developed a questionnaire as part of this study and it is not under a copyright more restrictive than CC-BY, please include a copy, in both the original language and English, as Supporting Information.

Information such as the patient's characteristics was acquired using a standard clinical form.

4. Please ensure you have thoroughly discussed any potential limitations of this study within the Discussion section, including the potential impact of confounding factors.

We have included all of the study's potential limitations, which are listed in lines 207-215.

 5.  We note that Figure S1 in your submission contain map images which may be copyrighted. All PLOS content is published under the Creative Commons Attribution License (CC BY 4.0), which means that the manuscript, images, and Supporting Information files will be freely available online, and any third party is permitted to access, download, copy, distribute, and use these materials in any way, even commercially, with proper attribution. For these reasons, we cannot publish previously copyrighted maps or satellite images created using proprietary data, such as Google software (Google Maps, Street View, and Earth). For more information, see our copyright guidelines: <http://journals.plos.org/plosone/s/licenses-and-copyright>.

Because we were unable to secure the appropriate authorization from the original copyright holder to publish that figure (S1), we opted to remove it in keeping with the alternatives (5.2) provided to us.

**Reviewer #1:**

**Comment 1:** While the study appears sound, the data presentation is flawed. Specifically, according to table 3, 258 of the susceptible patients have a history of past TB, while in the results it is stated the contrary (266 new and 17 relapses). As a result, after statistical analysis, the authors erroneously claim in line 160-161,
that "Newly diagnosed patients were more likely to have MDR than patients who had previously received treatment". Also in table 3, the number of MDR cases taken for statistical analysis is 8 for all variables. The study found a total of 19 MDR cases. It is not clear what the number 8 corresponds to; so is unclear the whole statistical analysis based in table 3.
I advise the authors to improve table 2 by putting only numbers of samples with mutations found for each of the mutations, instead of all (mutations found and not found). This will make the data presentation easier to follow. Also, in the table header please put mutation found/not found instead of susceptible/resistance.

Thank you for such a succinct overview and for bringing these concerns to our attention. The errors have been corrected, and table 3 has been amended, as highlighted in the revised manuscript. We also changed the statement (now lines 153-154) to now read, "... previously treated patients were more likely to have MDR than newly diagnosed patients ..." In addition, to make table 2 more understandable, we removed the column for “no mutations”. Table 2's header has also been amended to now read "Frequency of rpoB, KatG, and InhA mutations in Mycobacterium tuberculosis causing PTB..." as well as the 95% confidence interval has been added for interpretation purposes.

**Comment 2:** The authors use age groups 18-37, 38-57, 58-77, >=78. The WHO standard forms use other age groups (15-24, 25-44, 45-64, >=65. The authors may consider reanalysing their data according to these groups to better check for age-resistance correlations.

Following a new analysis in accordance with these recommendations, changes have been made to tables 1, 3, abstract (line 28) and in the discussion (line 202).

**Comment 3:** Line 30 contains a typo error. Please put 5.9% instead of 9.5%

Thank you for pointing that out. The error has been corrected.

**Reviewer #2:**

Surveillance for drug resistance TB is very important however the manuscript has many flaws that I have highlighted within the submitted manuscript. I also think the rationale for the work done is not well articulated and moreover the amount of work done is minimal. There will be much benefit if this method has been compared with at least one traditional method for DR determination. This will allow for comparison in terms of simplicity, sensitivity and specificity as well as cost.

Thank you for including such a concise overview and suggestions on how to improve this work. The primary goal of this study was to determine the levels of RIF and INH drug resistance in this location rather than evaluating the various techniques of assessing DR. We are aware that DST can be performed using a variety of techniques ranging from conventional to several molecular methods and we chose the qPCR-HRMA because various studies have shown that this technique has sensitivity and specificity of 94% to 99% respectively (1–3). We can, however, use this concept at a later date and conduct a comparison study of HRMA and another method in our setup in another study.

**Other comments**

1. The manuscript needs a through language review

Thank you for the observation. We have so far corrected the language to the best of our ability.

2. Lines 31-32: what is the reference data

Not only were our findings used as references, but so are the WHO guidelines. Nevertheless, that section of the abstract has been amended.

3. Lines 44-49: very long sentence, loosing meaning

The sentence has been paraphrased.

4. Lines 54-56: A major reason is that these are the backbone of TB treatment

Thank you for the suggestion and this has been incorporated into the sentence (now line 52)

5. Line 67: The authors indicated in the background that they find the methods, they used to assess DR as simple and straight forward. I do not agree that detection of mutations using melting curves is simpler in the Africa context.

While we acknowledge that HRMA is not the cheapest technology to use in a resource-limited setting like Uganda, multiple studies have shown that it is specific, sensitive, and quicker at detecting drug-resistant mutations. Since our primary goal was not to compare this approach (HRMA) to others techniques in terms of application and cost, but rather to determine the levels of drug resistance in this region we saw it paramount to inform readers of some of the different approaches used to screen for DR and support our reasons for using this technique since it is not the standard technique.

6. The authors should be mindful that the biomarkers (315 for KatG and -8 and -15 for inhA) that they only used to analyse for INH detects less than 90% of isoniazid resistance in Uganda. This was not discussed as a limitation of the study.
   PLoS One. 2018; 13(5): e0198091.Published online 2018 May 30. doi: 10.1371/journal.pone.0198091

Thank you for pointing this out and we have now included this as one of our study limitations.

7. The referenced article for the primers as indicated in the methodology section does not correspond to the cited article (Poudel et al. 2012). The cited article analyzed for DR conferring mutations among INH and RIF resistance phenotypes. There was no RT-PCR analysis nor the study designed primers for DR analysis.

Thank you for bringing this to our attention, we have now provided the primer sequence that were used in this study as a supplementary material (S1: Table 1).

8. I find setting P value less than 0.005 as indication of significance of difference in measured variable too high. I suggest the authors need to review the statistical analysis again. I think the burden of DR in TB is very crucial, that one needs to be critical in following leads

Thank you for pointing this out. This was a typo that has already been fixed, as shown at line 154. Thank you for the observation. The level of significance was at 0.05 and this was a typing error. As noted at line 158, this typo has been corrected.

9. The authors need to reduce the number of results repeated within the discussion. Also, the discussion has too many speculations

Thank you for drawing our attention to this. We addressed this issue by making changes to certain parts in the discussion section (green highlight)

**References**

1.	Araujo S, Goulart LR, Truman RW, Goulart IMB, Vissa V, Li W, et al. qPCR-High resolution melt analysis for drug susceptibility testing of Mycobacterium leprae directly from clinical specimens of leprosy patients. PLoS Negl Trop Dis. 2017;11(6):1–18.

2.	Galarza M, Fasabi M, Levano KS, Castillo E, Barreda N, Rodriguez M, et al. High-resolution melting analysis for molecular detection of multidrug resistance tuberculosis in Peruvian isolates. BMC Infect Dis [Internet]. 2016;16(1):1–6. Available from: http://dx.doi.org/10.1186/s12879-016-1615-y

3.	Arefzadeh S, Azimi T, Nasiri MJ, Nikpor Z, Dabiri H, Doustdar F, et al. High-resolution melt curve analysis for rapid detection of rifampicin resistance in Mycobacterium tuberculosis: a single-centre study in Iran. New Microbes New Infect [Internet]. 2020;35:100665. Available from: https://doi.org/10.1016/j.nmni.2020.100665

Thank you once more for your comments and we hope that we were able to resolve all of your concerns.

Sincerely,

Authors
