# Supplementary material for: Rifampicin and isoniazid drug resistance among patients diagnosed with pulmonary tuberculosis in southwestern Uganda
Source: PLoS One. 2021 Oct 29;16(10):e0259221. doi: 10.1371/journal.pone.0259221 (PMC8555815; doi:10.1371/journal.pone.0259221)
Supplement: Attachment — Submitted filename: Response to Reviewers.docx [file pone.0259221.s004.docx]

***Ref:*** ***Rifampicin and Isoniazid drug resistance among patients with pulmonary tuberculosis in southwestern Uganda***

Dear Dr. Richard Ente Claros
PLOS ONE.

We appreciate your time reviewing our work, and we have considered each feedback and made modifications where appropriate to address your concerns. The following is a thorough (in red) response to those comments;

1. Please upload a copy of Figure "S2. Fig. 1" which you refer to in your text on page 7. Or if the figure is no longer to be included as part of the submission, please remove all reference to it within the text.

Thank you for bringing this to our attention. There was an error in labelling the supplemental material. This has been corrected, and we now just have Table S1 (page 6) and Figure S1 (page 7), both of which have been uploaded.

2. Please upload a Response to Reviewers letter which should include a point-by-point response to each of the points made by the Editor and / or Reviewers. This should be uploaded as a 'Response to Reviewers' file type.

This reviewer’s response has been uploaded

Please follow this link for more information: [http://blogs.PLOS.org/everyone/2011/05/10/how-to-submit-your-revised-manuscript/](http://blogs.plos.org/everyone/2011/05/10/how-to-submit-your-revised-manuscript/)

3.  Please amend the title either on the online submission form or in your manuscript so that they are identical

The title on the online submission form has been amended to tally with title on the manuscript
